# Gait analysis after corrective surgery for adult spinal deformity - good sagittal balance with improved lumber lordosis is important

**DOI:** 10.1186/1748-7161-10-S1-O76

**Published:** 2015-01-19

**Authors:** Hideyuki Arima, Yu Yamato, Tomohiko Hasegawa, Daisuke Togawa, Sho Kobayashi, Tatsuya Yasuda, Yukihiro Matsuyama

**Affiliations:** 1 Department of Orthopedic Surgery Hamamatsu University School of Medicine Japan

## Objective

Patients with adult spinal deformity (ASD) commonly present with a forward trunk tilt when walking. We aimed to compare the trunk tilt while walking, before and after corrective surgery for ASD.

## Material and methods

We prospectively investigated 41 consecutive patients (mean age, 64 years; range, 22-81 years) who underwent corrective surgery for ASD from March 2011 to September 2012. The mean postoperative follow-up period was 26 (range, 15 - 33) months. Gait was analyzed before and 1 year after surgery, and the 4m walking status was recorded with a video camera. We measured the angle between the line connecting the greater trochanter and the pinna and a plumb line on the side during gait (gait-trunk tilt angle). We investigated the gait-trunk tilt angle (GTA) before and after corrective surgery. Patients were divided into two groups based on the postoperative GTA: Group A (28 patients; 0 - 10°) and Group B (13 patients, >10°). Differences in sagittal vertical axis (SVA), lumbar lordosis (LL), and pelvic tilt (PT) were examined in both groups.

## Results

The mean preoperative GTA was 13° (range, -4 - 60°). The preoperative GTA negatively correlated with preoperative LL (R=-0.45, P<0.01) and preoperative PT (R=-0.55, P<0.01). The mean postoperative GTA significantly improved to 8°(range, -5 - 33°, P<0.01). The postoperative GTA in Groups A and B improved from 8° to 4° and from 24° to 14°, respectively. The postoperative SVA in Groups A and B improved from 86 mm to 38 mm and from 149 mm to 115 mm, respectively. The postoperative SVA of Group A was significantly smaller than that of Group B (P<0.01). The postoperative LL in Groups A and B improved from 21° to 39° and from 17° to 28°, respectively. The postoperative LL of Group A was significantly larger than that of Group B (P<0.01). Patients with postoperative LL>40°, and postoperative SVA<50 mm were significantly more in Group A (P<0.01 and P<0.01, respectively).

## Conclusion

Gait analysis revealed that the forward trunk tilt angle while walking is associated with lumbar kyphosis and backward pelvic tilt. Patients with improved LL after corrective surgery could walk with good sagittal balance. Our results suggest that postoperative forward trunk tilt was improved when optimal correction of the spinal deformity was achieved.

